# A Case of Aneurism of the Right Femoral Artery Cured by Digital Compression

**Published:** 1859-01

**Authors:** Samuel W. Gross

**Affiliations:** Chief of the Surgical Clinic of the Jefferson Medical College, Philadelphia


ART. II.—*A Case of Aneurism of the Right Femoral Artery Cured by Digital Compression; with Remarks and a Statistical Report of twenty-two other cases treated by this method.* By SAMUEL W. GROSS, M.D., Chief of the Surgical Clinic of the Jefferson Medical College, Philadelphia.

ON the 1st of June, 1858, Sarah, a negress, thirty-two years of age, a native of Delaware, consulted Professor Gross on account of a pulsating tumor, seated at the upper and inner portion of the right thigh. She had always enjoyed good health. Her first symptoms appeared about ten months previously, when she experienced sharp shooting pains radiating through the hip, down the thigh to the knee, and extending as far as the ankle. These pains, which were not of long duration, were intermittent, occurring sometimes only once every two weeks, at other times every two or three days, being dependent, in some degree, upon the amount of exercise she took. She had been in the habit of rubbing her limb with vinegar and Cayenne pepper for these rheumatic pains, as she supposed them to be, when, in the latter part of March, she felt a small, hard lump at the spot adverted to, about the size of a walnut without its hull. This rapidly increased, and, about five and a half weeks ago, the pains in the tumor became so severe as to interfere with walking. On this account, and, inasmuch as the pain was relieved by the recumbent posture, she took to her bed.

Upon examination, a globular tumor was found at the upper and inner portion of the thigh, in Scarpa's triangle, about one inch and a half below Poupart's ligament. Its length was $4\frac{1}{2}$ inches and its width $5\frac{3}{4}$ inches. The circumference of the thigh was $26\frac{1}{4}$ inches; while the sound one measured only 23 inches. The tumor presented very distinct locomotion and a thrill at its superior portion. The *bruit de souffle* was well marked, and the pulsation was strong over the whole tumor, being distinctly felt by the patient, and 84 a minute. The sac could readily be emptied. Pressure on the artery above diminished the volume of the tumor and caused the sounds and impulse to cease. The arterial system was in other respects in a perfectly healthy condition, the heart's sounds being normal and the pulse perfectly regular.

The question in this case arose what plan of treatment should be employed. Although the woman was in good health, the operation of casting a ligature around the external iliac appeared too formidable, when compared with the safer mode afforded by compression. An instrument with a single pad, resembling Carte's circular compressor, was ordered to be made, the patient in the mean while being placed upon the use of purgatives, with low diet, and rest in the recumbent posture. The instrument, when finished, was found to be too small for the patient's thigh, and was therefore cast aside without the pad having been made to bear upon the artery at all. Not wishing to delay the treatment any longer, Prof. Gross placed the patient under my charge, compression by means of the fingers having been determined upon. Having obtained a suitable number of intelligent assistants, on Thursday morning, the 10th of June, at ten minutes after ten o'clock, a grain of sulphate of morphia having been administered, I began the treatment by applying the thumb over the artery as it passes over the pubic bone. At the end of an hour the temperature of the limb was somewhat diminished. At one o'clock the patient complained of sickness at the stomach, which was relieved by half a grain of morphia, and in an hour and a half she fell asleep. During the afternoon she felt drowsy and did not complain of pain.

11 P.M.—She has been complaining of pain for the last hour at the point of pressure, whenever the assistants relieved each other; in other words, when the pressure was relaxed. The knee is the seat of tingling pain, which extends to the toes, and the whole limb is painful when touched.

Friday, 10 A.M.—The tumor feels very solid and cannot be emptied of its contents. There is no bruit; but slight pulsation and thrill. The point of pressure has begun to be painful, and, to prevent any irritation of the skin, moistened flour was spread over it.

$5\frac{1}{2}$ P.M.—The tumor is very hard, but not diminished in size. No thrill or bruit, and but very feeble pulsation. The compressed spot is quite painful. Treatment discontinued after $31\frac{1}{4}$ hours' employment. A grain of morphia was administered, and pounded ice in a bladder was applied, to be continued during the night.

Saturday, 10 A.M.—The patient slept well last night. The tumor is softer, but cannot be emptied. The pulsations are stronger, and there is some thrill. The compression was renewed at twenty minutes to eleven o'clock, and in an hour she complained of great pain in the knee, which soon extended to the toes. At half-past twelve the patient took one grain of morphia; the pain soon ceased, but occasionally returned. At half-past five half a grain of morphia was administered.

At 8 P.M. the point of pressure gave so much pain that a thin opium plaster was interposed between the skin and finger to avoid more irritation. This had a good effect in palliating the suffering.

10 P.M.—The patient complains of much pain throughout the whole extent of the tumor. The thrill has disappeared, and the pulsations are becoming feeble. The femoral artery is beating forcibly, and a collateral branch, about the size of the ulnar artery, can be felt beating at the inner and posterior portion of the tumor.

At 11 P.M. half a grain of morphia was administered; faintest perceptible pulsations.

At $12\frac{1}{4}$ all pulsation had ceased. The femoral still pulsated pretty freely. The compression was continued for another hour, at the end of which time the femoral was beating gently.

At $2\frac{1}{2}$ o'clock, being satisfied that nothing more could be done, and that the circulation in the tumor was arrested, the patient was left, half a grain of morphia having been administered.

For a few days after this event the patient had slight tingling pains in the limb. Up to the time of her departure her appetite was good, and she slept well and was comfortable in every particular.

In about a week a gum ammoniac and mercurial plaster was placed over the tumor, and her diet was restricted. The tumor gradually diminished in volume, and she left for home on the 6th of July, not quite a month after the commencement of the treatment. At the time of her departure the affected limb had diminished to exactly the same size as the sound one. The femoral artery was pervious to within an inch above the tumor, and she could walk with comfort.

On the 13th of October, Sarah came to the city to report herself. The tumor had diminished to the size of a walnut and was very solid. The locomotion was perfect and her health was good in every respect.

*Remarks.*—This case is an admirable example of the cure of aneurism by digital compression, even when this has been interrupted. The total amount of time necessary for its cure was forty-five hours and fifty-five minutes: at the first sitting, thirty-one hours and twenty minutes; at the second, fourteen hours and thirty-five minutes.

The compression was almost total, preventing the blood from entering the tumor at all; and the whole time in which the blood did traverse the sac did not exceed two hours.

There cannot be a possibility of doubt, judging from the state of the tumor, that, had the compression at the first sitting been continued for two or three hours longer, a cure would have been the result. This was prevented, however, principally on account of the pain at the point of pressure, and also on account of the fatigue of the assistants and myself. I therefore preferred to wait, feeling confident that at the next meeting we would see the success of the treatment. My mind was also biased, when I remembered two cases reported respectively by Mr. Porter and Mr. Cusack, in both of which mechanical compression had been used, but proving irksome to the patients, was discontinued. The sacs were hard and incompressible, but pulsation still continued; the patients got out of bed and walked about, and yet deposition of fibrine continued until a cure was effected. The state of the tumor in the above case was exactly in the same condition as in those reported by the Dublin surgeons, and I expected, with the aid of the pounded ice, that a similar result would follow. In this, however, I was disappointed, and had to recommence the treatment.

The pressure was made mainly over the artery, where it crossed the horizontal ramus of the pubes; but when the pain became a source of annoyance, the finger was shifted and pressure was applied over the artery in the space between Poupart's ligament and where the saphenous joins the femoral vein. When made upon the former spot it was more easily kept up, and with less pain to the patient and to the finger of the operator. Throughout the whole course of the treatment the pain was not very great, owing to the free use of anodynes, which I consider in these cases of paramount importance to lessen arterial action, relieve suffering, and calm irritability of the system. An interesting point, in connection with other reported cases, is the establishment of the collateral circulation, as it will have been seen, from a perusal of the case, that a large arterial trunk pulsated in a very marked manner previous to the obliteration of the sac. At no time was the temperature of the limb much below that of the sound one, nor was there much œdematous swelling of the extremity, showing that the vein was not compressed. A point in favor of the compression not being altogether so forcible as entirely to prevent the blood from passing into the sac, is, that when it was total, the patient experienced more of that tingling pain than when the reverse was the case.

To Dr. Wood, and Messrs. Brinton, Glenn, McCondichie, Tomlinson, Blicke, Dysart, Purefoy, and Murfree, students of medicine in this city, much credit is due for their kind assistance and the praiseworthy zeal and interest which they manifested in the cure of this case.

Since the revival of the treatment of anuerism by compression, principally through the instrumentality of the Dublin surgeons, this mode of treatment has come into more general use, and its success is due to the better knowledge of the rationale of the mode of cure, and consequently the more effectual means employed for that purpose.

Although in the hands of a few eminent surgeons the ligature has been attended with results which, strange to say, have not been attained by others of apparently equal skill and care,—which good results have naturally led them to prefer that method to any other,—yet by most surgeons it has been discarded, until, at least, the more safe method has been put to test. There are still a few, who, instead of being guided by the more proper feeling of giving additional security and comfort to the patient, prefer the notoriety of a brilliant operation, gaining everything from the *éclat* dependent thereon, and occasionally not allowing the result of their treatment to be brought to light. The advantages of compression are so very obvious, that it is wonderful that it does not supersede all other methods of treatment. It is true there are cases in which the aneurism is so seated that this procedure would be out of the question, and the aneurism may be in such a condition as imperatively to call for the use of the ligature; but in all external aneurisms where pressure can be brought to bear upon the artery, it should unquestionably be employed. Conservatism in surgery is a term unknown in the vocabulary of some men, but those who do know it, and freely employ it, lead certainly a more quiet and conscientious, if not a more brilliant life. The advantages of compression over the ligature may be stated in a few words. It is a more simple, safe, and certain procedure, and, if persevered in, will almost always be successful. It is not followed by secondary hemorrhage. There is but little danger of erysipelas, suppuration of the sac, phlebitis, and gangrene. Constitutional effects are not so apt to succeed it. There is no danger of neuritis and purulent collections in the common sheath of the vessels. The length of time required for the cure, and the suffering are not so great. The mode of cure more closely resembles that effected by nature, and is apt to be more permanent, especially if the ligature be not applied at a distance from the sac; in cases where the ligature is near the sac, a loose coagulum may form and secondary aneurism may ensue. Lastly, and not the least reason of all, is that the surgeon does not feel that anxiety for his patient, as is always the case after a capital operation.

It is not necessary to adduce facts in substantiation of the above statements, for the records of all cases abundantly prove their validity.

As instrumental compression has such advantages over the ligature, so pressure exerted by the fingers possesses some advantages over that made by apparatus. Thus, it is quicker and less painful; it can be regulated better, and in some situations can be made to act upon the artery alone; it is applicable when apparatus is not; and in cases in which mechanical contrivances cannot be borne, it can be used to excite a tolerance of the skin previous to their employment.

The following are condensed reports of all the cases which have been treated by digital compression, employed either alone or in conjunction with apparatus. They are given in regular succession, in the order of the years in which they occurred:—*

* In an interesting communication made to the *Société de Chirurgie*, by Mons. Ar. Verneuil, and published in the *Gazette Hebdomadaire*, 30th October, 1857, p. 773, will be found the statistics of seventeen cases of aneurism, all popliteal but four, treated by digital compression. To this paper, which may also be found in the *Dublin Quar. Journ. of Med. Sci.*, May, 1858, p. 486, in the *Amer. Journ. of Med. Sci.*, July, 1858, p. 234, in the *New Orleans Med. and Surg. Journal*, Nov., 1858, p. 840, as well as to the work of M. Broca, I am indebted for many of the facts here presented. For further details of these cases, these two sources may be consulted.

*Case* 1.—In 1844, MR. E. GREATREZ was the first to employ digital compression, in a patient with popliteal aneurism of the right side. The compression was total and double; being made by an Italian tourniquet, alternating with the fingers of the patient. The compressor was placed over the artery below Poupart's ligament, and when the pain produced by it became unbearable, the patient relaxed the pressure for a few moments, at the same time applying his fingers very firmly to the artery just above the instrument, so as to allow of no blood passing through the tumor. The cure was effected in twenty-four hours. In this case, therefore, the fingers were but accessory to the instrument.

*Case* 2.—In 1846, M. VANZETTI employed digital compression alone, in a case of popliteal aneurism, by means of assistants, at the hospital of Kharkoff, in Russia. This was maintained for two days, but was inefficient. The patient was cured by the ligature.

*Case* 3.—In 1847, MR. TUFFNELL had, in a case of popliteal aneurism, been employing double and alternate pressure by apparatus. The upper pad giving rise to inflammation of the inguinal glands, the finger of the patient was substituted. This pressure was alternated with that of the instrument for twenty-four hours, when the inguinal pad was again used. The cure was effected in seven days.

*Case* 4.—In the same year, PROF. J. KNIGHT, of New Haven, had a case of popliteal aneurism, in which apparatus could not be borne at all. He cured the case in forty hours by *digital compression alone*, made by intelligent assistants, upon the femoral artery over the pubes.

*Case* 5.—In 1848, DR. W. H. VAN BUREN employed, for thirty-six hours, digital compression over the external iliac, in a case of true inguinal aneurism. Being unbearable, the artery was ligated and the patient cured.

*Case* 6.—One month later, in the same year, DR. GEORGE FOX, in a case of large inguinal aneurism, applied digital pressure to the external iliac for ninety-six hours. On account of the fatigue of the assistants, and being unable to procure more, the pressure was discontinued, the condition of the tumor being much improved. Mechanical compression was then employed for eight days, and the patient was finally cured by ligation of the external iliac.

*Case* 7.—PROF. WILLARD PARKER, in the same year, in a case of diffuse aneurism of the femoral artery consecutive to ligation, employed digital pressure for seventy-two hours with apparent success; but the pulsation having returned, compression was made by a weight in the groin for seven days, and a cure was effected.

*Cases* 8, 9.—In 1850, DR. J. R. WOOD treated two cases of popliteal aneurism by digital pressure on the pubes, alternating with Dupuytren's compressor about the middle of the thigh. In the first case the cure was effected in forty-eight hours, and in the second, in eleven hours and a half.

*Case* 10.—In 1851, MR. NORGATE employed digital compression, in a case of popliteal aneurism, for twenty-four hours, having previously used bad apparatus for five days. This was inefficient, and the femoral was ligated; the operation was followed by gangrene, necessitating amputation, but the patient recovered.

*Case* 11.—In 1852, MR. J. MONRO had a case of popliteal aneurism in which digital compression was kept up for three days by convalescent patients in the hospital. Mechanical compression was then substituted and a cure resulted in sixteen days.

*Case* 12.—In 1854, MR. WM. JAMESON employed digital compression for a popliteal aneurism for four hours, when it was discontinued on account of the patient being irritable and rebellious. Mechanical compression was substituted, and a cure was produced in six days.

*Case* 13.—In the same year, a patient, under the charge of MR. COLLES, cured himself, without assistance, of a diffuse popliteal aneurism, by intermittent digital compression, in seven days.

*Case* 14.—During the same year, and under the same surgeon, a femoral aneurism was cured in about six days, by partial, irregular, alternating digital and mechanical compression.

*Case* 15.—M. VANZETTI, in the same year, cured a popliteal aneurism, by digital compression, in forty-eight hours, various apparatus having been previously applied.

*Case* 16.—In 1855, M. NÉLATON, in a case of popliteal aneurism, tried mechanical pressure for sixteen days. This not succeeding, he substituted digital compression, which was maintained for ninety-four hours. The cure was not permanent. The limb was amputated, and the patient died of purulent infection.

*Case* 17.—In the same year, the same surgeon employed digital compression conjointly and alternately with direct compression, with apparatus, in a case of arterio-venous aneurism at the bend of the elbow. In several days the cure was effected.

*Case* 18.—In the same year, M. VANZETTI had an intelligent patient with a popliteal aneurism. Understanding the plan of treatment, which was deferred for twenty days, he himself, during this time, made pressure on the femoral artery, which seemed to have a slight influence upon the tumor. The treatment was confided to six intelligent assistants, and in four hours the patient was cured.

*Case* 19.—In 1856, M. MICHAUX employed intermittent mechanical pressure, in a femoral aneurism, for four days. Digital compression was then substituted, and in twenty-four hours the cure was complete.

| Number. | Operators. | Sex. | Age. | Date. | Variety of Aneurism. | Style of Compression. | Duration. | Result. | Remarks. | Authorities. |
|---|---|---|---|---|---|---|---|---|---|---|
| 1 | Edw. Greatrez.. | Male | 27 | 1844 | Popliteal | Total digital and instrumental. | 24 hours | Cure | The patient regulated the pressure. | Medico-Chirurgical Transactions, vol. xxviii. p. 39, 1845. |
| 2 | Vanzetti......... | ? | ? | 1846 | Popliteal | Dig., primary and alone. | 2 days | Inefficient | Ligature of the femoral. Cure. | M. Verneuil's paper. |
| 3 | Tuffnell.......... | Male | 27 | 1847 | Popliteal | Total digital and instrumental. | 7 days | Cure | Finger of patient alternating with apparatus. | Dublin Medical Press, vol. xxi. p. 305, 1849; Broca. *op. cit.* p. 808. |
| 4 | J. Knight........ | Male | 48 | 1847 | Popliteal | Digital, total, and alone. | 40 hours | Cure | First cure by this method unassisted by other means; apparatus having proved intolerable. | Boston Med. & Surg. Journal, May, 1848; Trans. Am. Med. Assoc., vol. i. p. 169, 1848. |
| 5 | Van Buren...... | Male | 25 | 1848 | Inguinal | Dig., primary and alone. | 36 hours | Unbearable | Ligature of external iliac. Cure. | New York Journal Med., vol. ii., new series, p. 168, 1849. |
| 6 | Fox............... | Male | 24 | 1848 | Inguinal | Digital. | 96 hours | Failure | Mechanical pressure for 8 days. Ligature of external iliac. Cure. | Am. Jour. Med. Sci., 1849, new series, vol. xviii. p. 377. |
| 7 | Parker........... | ? | ? | 1848 | Diffuse femoral. | Digital primary. | 72 hours | Inefficient | Aneurism consecutive to ligature of femoral for popliteal aneurism. Compression by weight in groin for 7 days. Cure. | Trans. Am. Med. Assoc., vol. ii., p. 228, 1849. |
| 8 | Wood ............ | Male | 30 | 1850 | Popliteal | Digital and instrumental. | 2 days | Cure | Digital compression on pubis alternating with Dupuytren's compressor on thigh. | New York Journal Med., vol. vi., new series, p. 304, 1851. |
| 9 | Wood ............ | Male | 33 | 1850 | Popliteal | Total dig. and instrumental. | 11½ hrs. | Cure | | |
| 10 | Norgate.......... | Male | 34 | 1851 | Popliteal | Digital. | 24 hours | Failure | Previously bad apparatus for five days. Ligation of femoral; gangrene; amputation. Cure. | Dublin Medical Press, vol. xxvii. p. 247, 1852. |
| 11 | Monro............ | Male | 23 | 1852 | Popliteal | Dig. primary. | 3 days | Failure | Made by patients for sixteen days; subsequently mechanical compression. Cure. | London Lancet, vol. i. p. 117, 1853. |

| | | | | | | | | | | |
|---|---|---|---|---|---|---|---|---|---|---|
| 12 | Jameson......... | Male | 45 | 1854 | Popliteal | Dig. primary. | 4 hours | Failure | Subsequent mechanical compression and in six days a cure. | Dub. Hospital Gaz., vol. i. n. 5, p. 72, April 1st, 1854. |
| 13 | Colles............ | Male | 36 | 1854 | Diffuse popliteal | Intermittent dig., primary and alone. | 7 days | Cure | Compression made by patient. | Dub. Hospital Gaz., vol. i. p. 97, May 1st, 1854. |
| 14 | Colles............ | Male | 32 | 1854 | Femoral | Irreg., alter., dig., and mechanical. | About 6 days | Cure | ................................ | Med. Times & Gaz., Dec. 30th, 1854; Ranking's Abstract, No. xxi. p. 102, 1855. |
| 15 | Vanzetti......... | Male | 36 | 1854 | Popliteal | Digital. | 48 hours | Cure | Various apparatus were tried for a long time before and given up. | M. Verneuil's paper. |
| 16 | Nélaton.......... | Male | 32 | 1855 | Diffuse popliteal | Digital. | 94 hours | Inefficient | Previous mechanical compression for sixteen days. Limb amputated; purulent infection. Death. | Broca., *op. cit.* obs. 45, p. 766. |
| 17 | Nélaton.......... | Male | 70 | 1855 | Arterio-venous at elbow | Dig. and other varieties. | Several days. | Cure | ................................ | Broca., *op. cit.*, p. 293, obs. 7. |
| 18 | Vanzetti......... | Male | 27 | 1855 | Popliteal | Digital, total, primary, and alone. | 4 hours | Cure | The patient himself made intermittent, imperfect, digital compression for twenty days. | M. Verneuil's paper. |
| 19 | Michaux......... | Male | 50 | 1856 | Femoral | Digital total. | 24 hours | Cure | Previously intermittent mechanical pressure for four days. | M. Verneuil's paper. |
| 20 | Michaux......... | Male | 57 | 1856 | Popliteal | Intermittent digital. | 7 days | Cure | Previously mechanical compression. Signs of serious disease of heart. | Bost. Med. & Surg. Jour., July 15th, 1858, p. 484. |
| 21 | Gherini.......... | Male | 27 | 1857 | Varicose at elbow | Dig., primary and alone. | $3\frac{1}{2}$ hours | Cure | ................................ | Med. Times & Gaz., May 8th, 1858; Omodei Annali, vol. clxiii. p. 99. |
| 22 | Michaux......... | Male | 52 | 1857 | Popliteal | Dig., primary and alone. | 24 hours | Cure | Previously bleeding, low diet and digitalis. Compression partial and total. | Lancet, Sept. 11, 1858, p. 202; from Jour. de Med. et de Chirurg. Prat., May, 1858. |
| 23 | S. W. Gross..... | Female | 32 | 1858 | Femoral | Dig., tot., pri. and alone. | 45 hours 55 min. | Cure | ................................ | North Amer. Med.-Chir. Rev., Jan. 1859. |

*Case* 20.—The same surgeon, in the same year, in a case of popliteal aneurism, first employed a tourniquet and then substituted digital compression. A cure was effected in seven days, the compression having been several times withheld.

*Case* 21.—In 1857, M. GHERINI employed digital compression, primarily and alone, in a case of varicose aneurism at the elbow. A cure was effected in three hours and a half.

*Case* 22.—In the same year, M. MICHAUX, in a case of popliteal aneurism, employed digital compression, primarily and alone, first partial and then total, and effected a cure in twenty-four hours.

For further convenience of reference these cases have been drawn up in the form of a table, from which it will be seen that fifteen were successful and eight unsuccessful. The number of the different varieties and the result are shown in the following table :—

| Variety. | No. of Cases. | Cure. | Failure. |
|---|---|---|---|
| Popliteal | 15 | 10 | 5 |
| Femoral | 4 | 3 | 1 |
| Inguinal | 2 | ... | 2 |
| Arterio-Venous | 2 | 2 | .. |
| | 23 | 15 | 8 |

*Cures.*—In *five* cases the digital compression was employed *primarily* and *alone.* Nos. 13, 18, 21, 22, 23.

In *four* cases digital compression succeeded after apparatus had been abandoned. Nos. 4, 15, 19, 20.

*Five* times digital compression was alternated with pressure by apparatus. Nos. 1, 3, 8, 9, 14.

*Once* digital compression succeeded when combined and alternating with apparatus and direct compression of the tumor. No. 17.

*Failures.*—In *six* cases digital compression was tried before any other means. Nos. 2, 5, 6, 7, 11, 12.

In *two* cases digital compression had been employed after apparatus had been abandoned. Nos. 10, 16.

After examining these unsuccessful cases, the question naturally arises, were they all good tests of this mode of treatment, and would it not have been possible to have cured several of them had the treatment been continued for a longer time ?

Of these eight cases, it is probable that in No. 7 the cure would have been complete, had the compression been kept up several hours longer; in Case 11, the pressure must necessarily have been imperfect, on account of its having been applied by convalescent patients in the wards of a hospital. In Case 12, it was abandoned after four hours, because the patient was rebellious. In Cases 5 and 6, the pressure was in one unbearable, and in the other no more assistants could be procured; in both, moreover, the aneurism was inguinal, where, on account of its unfavorable situation, it is exceedingly difficult to maintain pressure, and consequently a favorable result could scarcely be expected. Cases 2, 10, and 16, are the only ones which we would regard as perfect failures. In examining the ultimate results of these unsuccessful cases, we find that in four the artery was subsequently ligated, and all but one were successful, Nos. 2, 5, 6, 10; two were amputated, Nos. 10, 16; and one patient died of purulent infection. In three cases, subsequent mechanical compression resulted in a cure, Nos. 7, 11, 12. In Cases 7 and 12, the tumor was so modified as to render a cure by apparatus effectual. In Case 11, digital compression rendered apparatus bearable.

The length of time required for the cure of fourteen of these successful cases—No. 17 has to be rejected, as the period is so indefinite—averaged two days and two-thirds. When the compression was primary and employed alone, the average was two days and thirty minutes; when double and alternate, three days and seven hours; and when employed after apparatus had been abandoned, the mean duration was two days and twenty-two hours. Let us compare these results with those of aneurism treated by mechanical compression alone. Thus, Mr. Hutchinson found in the London hospitals that of twenty-six cases of femoral and popliteal aneurism cured by this method, the average duration was nineteen days, while in the Dublin cases the mean duration of treatment was twenty-five days.* M. Broca has found that in ninety-nine cases the length of time required for a cure was a little less than fifteen days.† At the present time we may therefore state that digital compression alone has effected cures in the shortest time. Next in order comes alternate digital and mechanical compression; and lastly, mechanical compression alone requires the longest time for a cure, although the duration of this plan is far less than that of the treatment by the ligature. The reason why mechanical compression has not succeeded in a shorter space of time, is on account of its mal-application in most cases; and when this point has been properly attended to, there can be but little doubt that a cure will result in half the time it will by the ligature.

A striking point in these twelve cures is, that five were effected by pressure with the finger alternating with the use of the apparatus. In two of these cases the pressure was regulated and kept up by the patients, Nos. 1, 3. In the remaining three, Nos. 8, 9, 14, the compression was made

* See Erichsen's Surgery, second edition, pp. 522–23; London, 1857.

† Broca des Anéurysmes, etc., p. 844; Paris, 1856.

by assistants. As this plan is so simple, has been attended with the best results, affords such relief to the patient and operator, and has always effected a cure whenever employed, it should be preferred to all other methods.

The case of Mr. Colles, No. 13, is interesting from the fact that the patient cured himself in seven days, by irregular intermittent digital compression, the treatment being carried on without the knowledge of the surgeon.

In M. Vanzetti's case, No. 18, the patient had kept up the pressure himself for twenty days previous to the interference of the surgeon, so modifying the tumor as to render the cure perfect in four hours.

Thus it will be seen that in a little more than one-fourth of these cases the patients had been, in a greater or less degree, instrumental in the cure, leaving the surgeon but little to do.

Another interesting fact is, as M. Broca observes, "that digital compression is an American procedure, and the principal merit thereof belongs, undoubtedly, to Prof. Knight," who was the first to cure a case by this method, unassisted by apparatus. Besides his case, six others have been operated on in this country, making seven of the twenty-three cases; four of which were successful.

Although the first cure was effected in this country, the case was the second that had been treated by digital compression alone, the first having been in the hands of M. Vanzetti, Professor of Surgery in the University of Padua, who did not, however, succeed in curing his patient. The priority belongs, therefore, to the latter gentleman, who has, moreover, applied this method in four cases of external phlegmonous inflammations, with happy results.

The compression should, in our opinion, in cases of popliteal and femoral aneurism, when not contra-indicated, be made upon the femoral artery as it lies over the pubic bone. At this point it is more easy of application, and causes less pain to the patient and fatigue to the operator. If made a little lower down, the inguinal glands are apt to become inflamed and give rise to great suffering. The vein, moreover, is here on the inside of the artery, and if the pressure be made over the course of the artery, it alone will be compressed. Throughout the whole extent of the rest of the thigh, the vein will be sure to be involved.

We trust that this plan of treatment may be more generally adopted than it has been heretofore. It is always possible to obtain a sufficient number of assistants in private and hospital practice, who would be but too glad to avail themselves of such rare opportunities for observation, and do all in their power to lessen the dangers and sufferings of a fellow-being. Its great value certainly calls for repeated trials; let them be made and let new facts be added to those already presented, so that we may more readily arrive at the comparative value of all the modes of treatment of this class of cases.

From all the data to be found in these cases, we may deduce the following propositions:—

I. Digital compression, uncombined with apparatus, was first attended with success in the hands of Dr. Knight; but to M. Vanzetti is due the merit of having first introduced it into practice.

II. It has never been followed by bad consequences, and when not successful, it so modifies the tumor and the collateral circulation as to render a cure by other means almost certain.

III. It has been employed alone, either previous or subsequent to mechanical compression, in fourteen instances, eight being failures.

IV. In only seven cases has it been employed primarily and alone, and in all but two with perfect success.

V. When double and alternating, it has effected cures in every case, five in number, and therefore deserves special attention.

VI. In most of the cases the compression has been total; but this is not necessary for a favorable result.

VII. It has effected cures, whether it was continued, interrupted, or intermittent; in some cases the patient applying the pressure.

VIII. When properly employed and continued for a sufficient length of time, and the cases are suitable ones, it can scarcely fail to accomplish a cure. Inguinal aneurisms are not fit cases for this procedure.

IX. It is less apt to give rise to inflammation of the integument, and has been borne when mechanical pressure has produced an eschar.

X. It can be used when apparatus has failed or is intolerable. In a majority of these cases, cures have been accomplished.

XI. In certain situations it can be made to bear upon the artery alone. It is far less painful and requires a much shorter time for a cure than any other method of treatment.

